# Advances in Childhood Diabetes

**DOI:** 10.3390/children12091195

**Published:** 2025-09-08

**Authors:** Zvi Laron

**Affiliations:** Endocrinology and Diabetes Research Unit Schneider Children’s Medical Center, Tel Aviv University Medical School, Petah Tikva 49200, Israel; laronz@clalit.org.il; Tel.: +972-522545280

Despite great technological advances in Type 1 Diabetes (TID) [1] the progressive increase in the incidence worldwide has not been arrested [2,3]. There is evidence that environmental factors such as viruses have an important role in its etiology [4] and incidence.

In this Special Issue we attempted to include articles related both to the incidence and etiology of Type 1 autoimmune diabetes and neonatal diabetes as well as methods to improve their management. Blumenfeld et al. [5], studying the incidence of TID before and during the COVID-19 pandemic, registered a significant increase in the incidence before the vaccination and a decrease following the introduction of the COVID-19 vaccination. Despite this finding, the authors conclude that no clear link between COVID-19 and etiology could be drawn.

Kennedy et al. [6] reviews the procedure to measure beta cell reserve as an indicator for intervention in the autoimmune of TID by new drugs.

Three articles relate to the management of TID. Zahran et al. [7] reviews the diagnosis and management of the hyperosmolar syndrome, a serious biochemical complication resulting from delayed diagnosis. They stress the importance of education of health care personnel to avoid this pathological state.

Two studies in this Special Issue relate to psycho-social aspects. Villaecija et al. [8] studied the psychometric properties of a diabetes self-management test in a large group of adolescents and found it helpful in the management of the disease. A similar study with the same conclusions but using another method, including videos to evaluate the quality of self-management, was conducted by Kime et al. [9] in the UK.

The last article is a patient report of a two and a half month baby with neonatal diabetes [10]. The authors discuss the diagnosis including genetic analysis and management.

We hope the content of this Special Issue will be helpful to all of those caring for children with diabetes.
